# Meat Diet

**Published:** 1890-02-08

**Authors:** 


### DIETETICS.

### MEAT DIET.

We referred to the subject of meat diet in the "Retrospect" of December 14th last, and in corroboration of what was then advanced regarding the possibility of persons living on meat diet alone, we note the following: Dr. Good, Professor of Clinical Medicine, Manitoba Medical College, states (*Lancet*, January 4th) that in the Mackenzie River region persons in the employ of the Hudson's Bay Company habitually live on meat alone, or on fish alone. Those living on fish alone enjoy rather the better health. But all are singularly free from constipation, and from digestive troubles. No vegetables of any kind are used. They are allowed one pound of flour in the year, which is consumed at Christmas in the shape of puddings. The life led by these men is one of great activity. Two are seventy years old, and in the enjoyment of a green old age. It is also remarked by Dr. Good that scurvy is quite unknown, although prevalent at another station named "York Factory." Here the diet consists largely of geese, the birds appearing in the spring, when they are shot and salted for winter use. At this station, the majority being clerks, the same active life is not led, which may be another element in the development of scurvy. Quite sufficient has now been advanced on this subject to show the necessity for a re-investigation of the accepted opinions regarding human food. It appears that the views promulgated in text books that man cannot live on a meat diet alone, because the human digestive organs are unequal to the task of digesting sufficient meat, must now be taken *cum grano salis*.

